# Economic Evaluation and Techno-Economic Sensitivity Analysis of a Mass Integrated Shrimp Biorefinery in North Colombia

**DOI:** 10.3390/polym12102397

**Published:** 2020-10-18

**Authors:** Antonio Zuorro, Kariana Andrea Moreno-Sader, Ángel Darío González-Delgado

**Affiliations:** 1 Department of Chemical Engineering, Materials & Environment, Sapienza-University of Rome, Piazzale Aldo Moro, 00185 Rome, Italy; 2 Chemical Engineering Department, Nanomaterials and Computer Aided Process Engineering Research Group (NIPAC), University of Cartagena, Avenida del Consulado St. 30, Cartagena de Indias 130015, Colombia; kmorenos@unicartagena.edu.co

**Keywords:** economic evaluation, techno-economic sensitivity, biorefinery, shrimp, chitin, chitosan, astaxanthin

## Abstract

The high freshwater consumption requirements in shrimp biorefinery approaches represents one of the major drawbacks of implementing these technologies within the shrimp processing industry. This also affects the costs associated with the plant operation, and consequently, the overall economic performance of the project. The application of mass integration tools such as water pinch analysis can reduce frewshwater consumption by up to 80%, contributing to shrimp biorefinery sustainability. In this work, the economic evaluation and the techno-economic sensitivity analysis for a mass integrated approach for shrimp biorefinery were performed to determine the economic feasibility of the project when located in the North-Colombia region and to identify the critical techno-economic variables affecting the profitability of the process. The integrated approach designed to process 4113.09 tons of fresh shrimp in Colombia reaches a return on investment (%ROI) at 65.88% and a net present value (NPV) at 10.40 MM USD. The process supports decreases of up to 28% in capacity of production and increases of 12% and 11% in the cost of raw materials and variable operating costs without incurring losses, respectively. These findings suggest that the proposed design of the water recycling network coupled to a shrimp biorefinery approach is attractive from an economic point of view.

## 1. Introduction

Shrimp represents approximately 45% of the total seafood consumed worldwide owing to its nutritional value and taste [1]. The current production of shrimp is estimated at 5.03 million tons per year with a growing market size [2]. The processing of shrimp, driven by its increasing demand, generates a large amount of waste such as shrimp heads and shells; the latter is composed of chitin, protein, minerals, and carotenoids [3]. Chitin is the second most available biopolymer in nature [4], which is considered an important material due to its properties such as biodegradability, non-toxicity, thermal stability, immunogenicity, and biocompatibility [5]. This biopolymer is widely used in papermaking [6], pharmaceutical [7] and cosmetics [8] industries, wastewater treatment, and agriculture [9]; however, it receives special interest as a precursor of chitosan [10].

Chitosan is the N-acetyl derivative of chitin, a natural polymer obtained after the alkaline deacetylation of chitin [11]. This biopolymer is characterized by its soluble, biodegradable, biocompatible, bioadhesive, antibacterial, hydrating, renewable, non-toxic, anti-allergic, and absorption properties [12]. The physicochemical characteristics of chitosan depend on the deacetylation degree, the solution viscosity, the drying temperature, and the percentage of acid solution [13]. Also, a large variety of chitosan derivatives exist; oligochitosan and N-carboxymethyl chitosan are useful water-soluble derivatives of chitosan which are produced by acid hydrolysis, enzymatic hydrolysis, or oxidative degradation of chitosan [14].

The shrimp exoskeleton is also an important natural source of carotenoids, especially astaxanthin, which is a type of xanthophyll responsible for the orange-red color of crustaceans [15]. Astaxanthin is an antioxidant ten times more effective than several other carotenoids, and consequently, it is considered the most valuable for food and pharmaceutical applications [16]. This carotenoid has been used mainly for tumor treatment [17], nutritional supplements [18], and as a food additive for the fishing industry [19].

The production of these added-value products from shrimp wastes along with the conventional processing of shrimp represents an attractive alternative towards a more responsible consumption of resources. To this end, a biorefinery approach can be applied to reduce the environmental impacts of shrimp production while increasing project profitability. The design of a shrimp-based biorefinery incorporating optimization techniques can also reduce the consumption of fresh material and decrease the waste generation rate [20], leading to a more sustainable process. Recent work reported better performance indicators for algae-based biorefineries when recovering wastewater. For the mass integrated approach, the authors found that the minimum selling price of the fuel can be reduced up to 150% compared to the non-integrated case without generating losses [21]. For an optimized process for the production of the biojet-fuel intermediate from biomass, reductions in total annual costs up to 89.76% were shown [22].

Several biorefinery configurations have been evaluated from an economic point of view, including an African palm biorefinery [23] and a lignocellulosic multi feedstock biorefinery [24]. These works considered economic indicators such as net present value, return on investment, and payback period, showing that both approaches were profitable with high sensitivity to variations in techno-economic parameters. For a mass integrated biorefinery approach to produce chitin, chitosan, astaxanthin, and shrimp meat, no information is available regarding its economic feasibility. Based on the current body of knowledge, it is necessary to perform an economic evaluation of the proposed approach to estimate project viability when design improvements are addressed using mass and heat integration tools.

This work aims to assess the economic performance of an integrated approach for a shrimp biorefinery to determine the profitability of the project when applying mass integration techniques (water pinch analysis). An economic sensitivity analysis is also performed to evaluate the effect of changes in production capacity, raw material costs, variable operating costs, and selling price of the products.

## 2. Materials and Methods

### 2.1. Process Description

**Figure 1:**
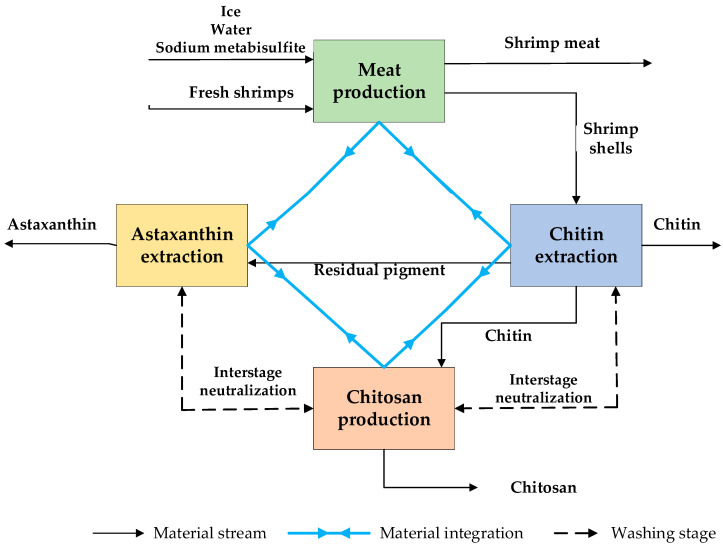
Process diagram of a mass integrated biorefinery based on shrimp.

As shown in Figure 1, the proposed shrimp biorefinery approach coupled with a direct water recycling network includes four main steps: meat production from conventional processing of fresh shrimp, chitin recovery, chitosan production, and astaxanthin recovery. The network was designed using pinch analysis to reduce the consumption of water up to 80% while recycling a fraction of sodium hydroxide and hydrochloric acid solutions. Fresh shrimps feed into the meat production unit composed of pretreatment stages, classification, and head and shell removal. The shrimps are initially washed at low temperatures with sodium metabisulfite to remove impurities and prevent shrimp melanosis [25]. The stained and damaged material is separated from the mainstream using a sorting stage. The heads and shells are also removed to obtain the shrimp meat under market specifications.

Following the experimental methodology proposed by Bonfante et al. [26], shrimp shells were cleaned up with water for organic impurities removal. The resulting stream enters into a drying stage, and a further crushing stage for particle size reduction to 0.5 mm. The carotenoid content is separated from the chitin-rich stream via the solvent extraction technique. To this end, a depigmentation stage is incorporated within the shrimp biorefinery approach to remove astaxanthin using 85% vol. ethanol [26]. The residual pigment stream undergoes two main stages in the astaxanthin recovery unit: solvent removal and evaporation. The former encompasses the separation of astaxanthin from ethanol using 10% vol. acetone that serves as a dragging agent [27], followed by centrifugation. The latter is performed at temperatures below 45 °C to remove the remaining solvent content.

The depigmented material is sent for demineralization, where minerals such as carbonates are removed using 1.5 M hydrochloric acid solution. This stage is required to avoid the hydrolysis of chitin in further processing [28]. The main stream feeds into an interstage neutralization process using 1 M sodium hydroxide solution and further washing [29]. The resulting wastewater containing NaOH is mixed with the freshwater stream and sent back for neutralization and washing according to the proposed water recycling network. Afterward, deproteination reactions take place when amino acids in shells contact with the sodium hydroxide solution at 1 M [30]. A nitrogenous extract results from this stage as a by-product with potential applications as fertilizer. The chitin-rich stream leaving the deproteination stage is neutralized using HCl solution at 1.5 M and washed thoroughly [29]. The wastewater from the chitin washing stage is also recycled to the previous interstage neutralization and washing (water recycling network) process. The chitin stream is split into two equal parts: one is sent to the chitosan production unit, while the remaining is dried above 60 °C and stored as a final by-product.

Chitin is transformed into chitosan through the removal of acetyl groups during the deacetylation reaction at 100 °C given as follows [31]:

$$D-N-\mathrm{Acetylglucosamine}+N_{a}\mathrm{OH}\to \mathrm{Chitosan}+C_{2}H_{3}N_{a}O_{2}\;\left(\mathrm{Reaction}\;1\right)$$

**Figure 2:**
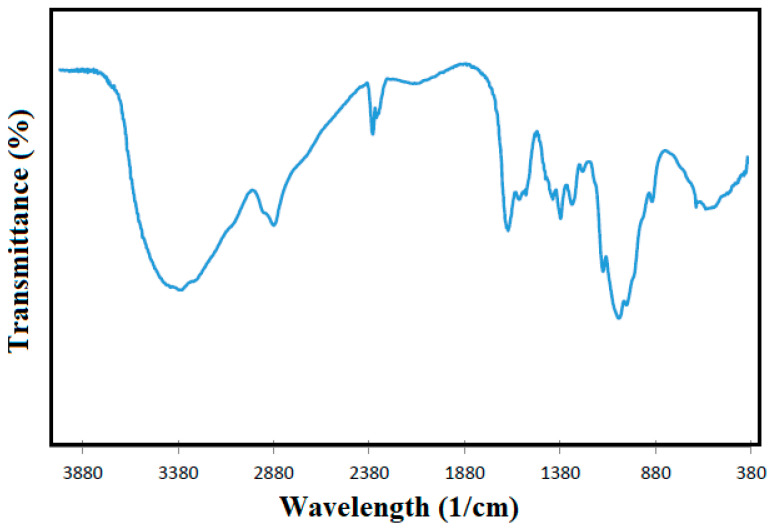
FTIR spectra of chitosan from shrimp shells.

In this stage, sodium hydroxide solution at 50% w/v is employed with ratio chitin to solution of 1:10 % *w/v*. The resulting chitosan is sent to neutralization with HCl solution at 1.5 M and washing; the wastewater rich in hydrochloric acid from this washing unit is recycled to the process following the proposed water network. This by-product is finally dried in an oven at 100 °C and stored for further selling [32]. The quality of chitosan is measured by the deacetylation degree (DA) at the laboratory scale, which represents the proportion of acetylglucosamine units in the polymer [33]. Figure 2 depicts the Fourier-transform infrared (FTIR) spectrum of chitosan, whose characteristic peaks are required to quantify the DA. The presence of absorbance peak around 1470–1620 cm^−1^ corresponded to amide bands I to III, and its relationship with a reference band at 1420 cm^−1^ was used during DA measurement [26]. A middle deacetylation degree around 81.81 was obtained for chitosan from shrimp shells similar to those reported for commercial chitosan.

Table 1 lists mass flowrates and operational conditions for the main process streams. For a processing capacity of 4113.09 t/y based on the shrimp production rate in North Colombia by 2018 [34], the proposed approach reached a production rate of 2417.66 t/y shrimp meat, 35.13 t/y chitin, 29.21 t/y chitosan, 99.55 t/y nitrogenous extract, and 1 t/y astaxanthin. The shrimp meat represents 93.62% of the products obtained; while chitin is 1.36%, chitosan 1.13%, astaxanthin 0.04%, and nitrogenous extract 3.85%. Besides, the recycling of wastewater within the water network minimized the overall consumption of freshwater, NaOH, and HCl.

### 2.2. Economic Evaluation

The economic analysis was used to evaluate the profitability of an integrated approach for shrimp biorefinery under key performance indicators [35]. Primary costs encompass Total Capital Investment (TCI) and Operating Costs (OC). The TCI is given by Equation (1) as a sum of three terms: Fixed Capital Investment (FCI) refers to the money needed to pay for equipment, piping, electrical installations, land, civil structures, legal costs, and control systems; Working Capital Investment (WCI) is the money necessary to pay for operating costs before the sale of products begins; Start-Up Costs (SUC) considers legal, publicity and employee training costs. The operating costs include Direct Production Costs (DPC), Fixed Charges (FCH), Plant Overhead (POH), and General Expenses (GE) [36], as shown in Equation (2).

(1) TCI=FCI + WCI + SUC

(2) OC=DPC + FCH + POH + GE

According to Equation (3), on-stream efficiency was calculated as the ratio between production capacity on break-even point BEP $\left(m_{BEP}\right)$ and the maximum production capacity $\left(m_{max}\right)$. Economic indicators such as gross profit (depreciation not included) (GP), gross profit (depreciation included) (DGP), profit after taxes (PAT), normalized variable operating costs (NVOC), economic potentials (EP1, EP2, EP3), cumulative cash flow (CCF), payback period (PBP), the return on investment (ROI%) and net present value (NPV) were calculated by Equations (4)–(13) [36].

(3) $$\eta_{On-stream}^{BEP}=\frac{m_{BEP}}{m_{max}}$$

(4) $$DGP=\underset{i}{\sum }m_{i}C_{i}^{v}-TAC$$

(5) PAT=DGP(1−itr)

(6) $$NVOC=\frac{AOC-FCH}{m_{RM}}$$

(7) $$EP_{1}=\underset{i}{\sum }m_{i}C_{i}{}^{v}-\;\underset{i}{\sum }m_{j}C_{j}{}^{RM}$$

(8) $$EP_{2}=\underset{i}{\sum }m_{i}C_{i}{}^{v}-\;\underset{i}{\sum }m_{j}C_{j}{}^{RM}-U$$

(9) $$EP_{3}=\underset{i}{\sum }m_{i}C_{i}{}^{v}-\;AOC$$

(10) $$CCF=\frac{\sum_{i}m_{i}C_{i}^{v}-AOC}{TCI}$$

(11) $$PBP=\frac{FCI}{PAT}$$

(12) $$\%ROI=\frac{PAT}{TCI}x100$$

(13) $$NPV=\underset{n}{\sum }ACF_{n}{\left(1+i\right)}^{-n}$$

where $m_{i}\;and\;C_{i}^{v}$ are the flowrate and selling price of product *i*, respectively, TAC is the total annualized cost, itr is the income tax rate, $m_{RM}$ is the raw material flowrate, $C_{j}^{RM}$ is the cost of raw material *j*, U are the utilities, $ACF_{n}$ is the net income for the *n*th year, and i the interest [37].

## 3. Results and Discussion

### 3.1. Economic Evaluation

The economic assessment for the mass integrated approach for a shrimp biorefinery was performed considering the assumptions shown in Table 2. The cost of raw materials and the selling price of products were estimated by vendor quotes from the Alibaba website [38]. Table 3 lists the selling price of the main product (shrimp meat) and the by-products (astaxanthin, chitosan, chitin, and nitrogenous extract).

The total capital investments for the mass integrated biorefinery based on shrimp were calculated by Equation (1), and the results for each term are shown in Table 4. The Fixed Capital Investment (FCI) was calculated considering the costs associated with the following factors: purchase and installation of equipment, instrumentation, piping, electrical installations, buildings, services facilities, land, yard improvements, engineering and supervision, construction expenses, legal expenses, contractors’ fees, and contingency. The costs associated with the purchase of the equipment were determined using the Process Economics Analyzer tool from the Aspen Plus^®^ software. The Working Capital Investment (WCI) and Start-up Costs (SUC) are calculated as 50% and 10% of the FCI, respectively, according to Peter et.al. [39].

The results for annualized operating costs (OC) are summarized in Table 5. The direct production cost included the cost for raw materials, utilities, maintenance and repairs, operating supplies, operating labor, direct supervision and clerical labor, laboratory charges, and patents. The fixed charges involved the depreciation, local taxes, insurance, and interest. Plant overhead included expenses associated with hospital and medical services, general engineering, security services, recreation, cleaning, communications, transportation, and delivery. Overheads were calculated considering the money to cover administrative expenses, distribution costs, marketing, and research. All costs were estimated according to the actual value in Colombia. Table 6 summarizes the primary costs and annualized revenues for the mass integrated biorefinery based on shrimp.

### 3.2. Economic Indicators

Table 7 summarizes the economic indicators for the proposed approach. The return on investment (%ROI) calculated at 65.88% reveals the economic feasibility of the project, considering that projects with %ROI above 10–15% are feasible from the economic viewpoint [40]. However, the result of cumulative cash flow indicated that the initial investment is significantly higher compared to the annual revenues, and according to the payback period after depreciation, 6 years are required to recover the whole investment. These results are acceptable considering that the plant life is 15 years; at the end of the project, a net profit of 10.40 MMUSD is reached as indicated by the net present value.

Comparing with the economic results for other biorefineries it was found that the return on investment for a lignocellulosic multi feedstock biorefinery was 32% [24], and for a combined palm and jatropha biomass biorefinery for biodiesel and hydrogen production was 33.18% [41], which shows that the mass integrated approach for shrimp biorefinery is more economically attractive. The net profits for the biorefinery were estimated to be up to 95% higher than the net profit obtained in the chitosan production process from shrimp exoskeleton [42]. These findings revealed the attractiveness of incorporating by-products extraction units along with the chitosan synthesis from chitin under the biorefinery concept, in agreement of similar approaches [43] on agro-industrial residues [44]. Moreover, same savings could come from a better optimization of the process, either in terms of design [45], either in modelization [46] or extraction [47].

### 3.3. Sensitivity Analysis

**Figure 3:**
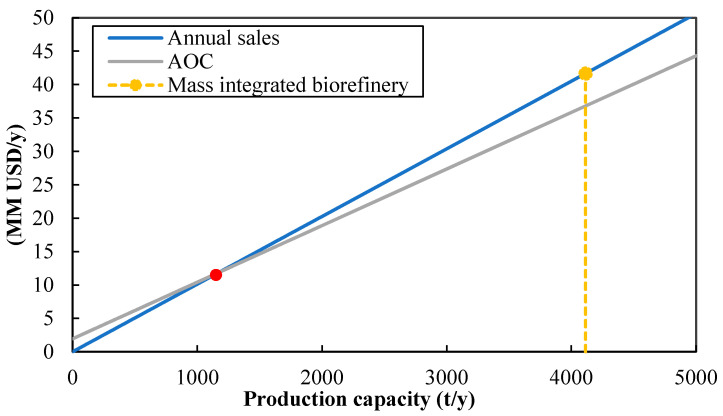
Break-even analysis for the mass integrated approach for a shrimp biorefinery.

The break-even analysis is illustrated in Figure 3. The process proved to be feasible from a techno-economic point of view by operating at 100% of the installed capacity since the annual sales are higher than the annual operating costs (AOC). The break-even point is achieved by processing 1150 tons of raw material per year, approximately 28% of the installed capacity. Therefore, the process can tolerate changes in the capacity of production, being beneficial given that the availability of fresh shrimp may depend on external factors such as climate and market conditions. According to these results, the production capacity can be reduced to less than half, and the process remains in the feasibility region.

**Figure 4:**
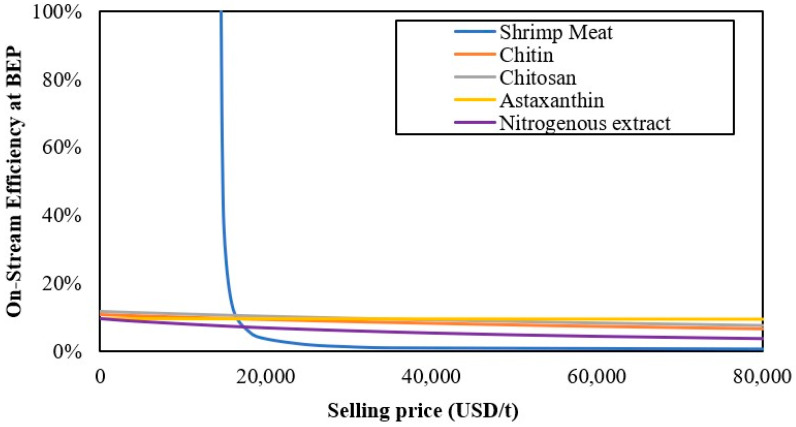
The effect of the selling price on on-stream efficiency.

The on-stream efficiency sensitivity analysis for the mass integrated biorefinery approach is shown in Figure 4. It can be shown that the on-stream efficiency is highly sensitive to changes in the selling price of shrimp meat, while the selling price of chitin, chitosan, nitrogenous extract, and astaxanthin has no substantial effects on the on-stream efficiency. Three regions can be identified in the figure: the first region where the on-stream efficiency presents a high sensitivity to the selling price; the second region, named the transition period, in which changes in on-stream efficiency are gradual, allowing for greater operability when varying market trends; and the third region, where the on-stream efficiency remains constant, disregarding the selling price of products. According to Table 3, the process is located in the second region; however, the current selling price of shrimp meat is near its critical value (16,000 USD/t) of moving towards unprofitability. It was also found that the selling price of the meat does not support decreases higher than 500 USD/t.

**Figure 5:**
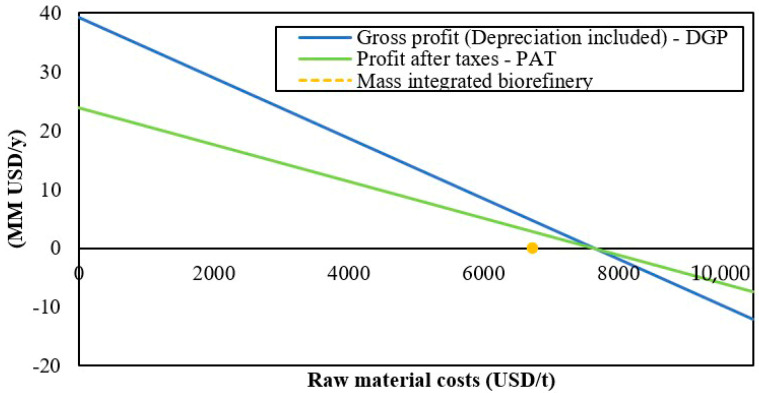
The effect of raw material costs on profitability.

The effect of raw material costs on the process profitability was also evaluated and the results are shown in Figure 5. The biorefinery describes a high sensitivity to changes in raw material costs with a critical point around 7600 USD/t; above this value, the process generates economic losses. According to Table 5, the current cost of raw materials is 6724.17 USD/t, which is an acceptable value because it can increase up to 12% without affecting the profitability of the project.

**Figure 6:**
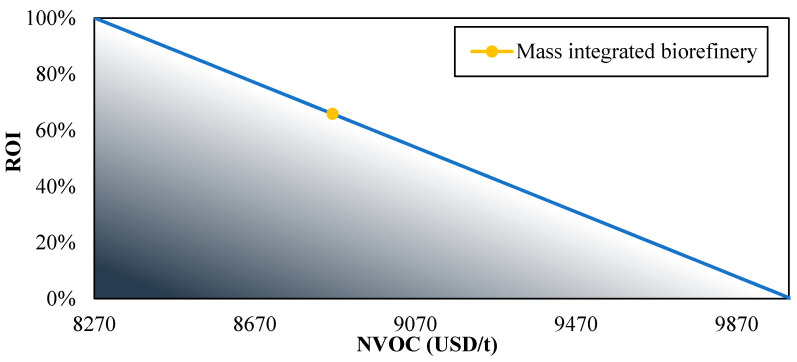
The effect of variable operating cost on return on investment.

**Figure 7:**
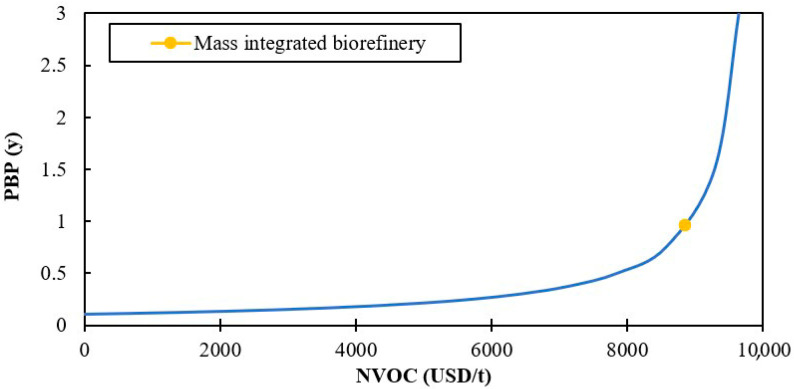
The effect of variable operating cost on the payback period.

Figure 6 and Figure 7 show the effect of variable operating costs on the return on investment and payback period, respectively. These findings showed that the NVOC reaches a critical value around 10,000 USD/t where the %ROI is null and the PBP tends to infinity. The variable operating costs for the biorefinery are approximately 11% below this value, indicating that the process can support slight increases. These results are favorable considering several common problems that can affect the NVOC, such as employee strikes, increased labor costs, and fuel supply. When variable operating costs are negligible, the process reaches ROI greater than 500% and a PBP less than a year. Similar projects such as a chitosan production process from shrimp exoskeletons and a plant to obtain agar from red algae showed a maximum return on investment of 34% [48] and 276% [35], respectively, when the NVOC = 0, indicating that mass integrated biorefinery based on shrimp presents a better performance in terms of the return on investment.

**Figure 8:**
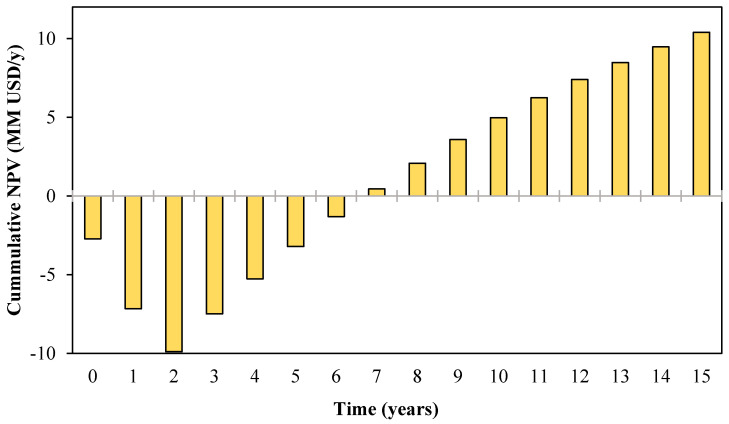
The net present value for the mass integrated approach for shrimp biorefinery.

The trends of the net present value during the 15 years of plant life are depicted in Figure 8. The techno-economic sensitivity analysis for the integrated approach showed a positive NPV after seven years. This project reaches NPV = 10.40 MM USD by the end of the project, yielding around three times greater than for the non-integrated biorefinery. Since a higher NPV should be selected, the incorporation of a water recycling network to reduce freshwater savings by up to 80% makes the implementation of a shrimp biorefinery more attractive for investors. 

## 4. Conclusions

The economic evaluation and techno-economic sensitivity analysis for a mass integrated approach for the production of shrimp meat, chitin, chitosan, nitrogenous extract, and astaxanthin under a biorefinery concept were carried out to determine its feasibility and to identify the critical techno-economic variables that affect the profitability of the process. For a processing capacity of 4113.09 t/year of fresh shrimp, the process is economically attractive, reaching %ROI at 65.88%, and 6 years are required to recover the whole investment. The proposed approach supports decreases by 28% in production capacity and increases up to 12% and 11% in the cost of raw materials and variable operating costs, respectively, without significant losses. The development of a mass integrated biorefinery was found to be attractive for coupling a water network within the design of a shrimp-based approach aiming to reduce freshwater and neutralization agent consumption.

## Figures and Tables

**Table 1:** Main process streams for mass-integrated biorefinery based on shrimp.

Streams	Fresh Shrimp	Shells	Meat	Astaxanthin	Nitrogenous Extract	Chitin	Chitosan
Temperature (K)	286.38		282.15	298.15	363.15	373.15	298.16
Pressure (kPa)	101.32	101.32	101.32	101.32	101.32	101.32	101.32
	Components mass flow (kg/h)						
L-Alanine	16.64	1.76	9.75	0.00	1.76	0.00	0.00
L-Glutamic-acid	28.68	3.04	16.81	0.00	3.04	0.00	0.00
L-phenylalanine	10.80	1.15	6.33	0.00	1.15	0.00	0.00
Methionine	9.70	1.03	5.68	0.00	1.03	0.00	0.00
Lysine	31.35	3.32	18.37	0.00	3.32	0.00	0.00
Calcium Carbonate	8.31	1.40	2.83	0.00	0.00	0.00	0.00
Calcium phosphate	20.86	3.52	7.11	0.00	0.00	0.00	0.00
Sodium carbonate	4.24	0.72	1.44	0.00	0.00	0.00	0.00
Magnesium carbonate	2.45	0.41	0.83	0.00	0.00	0.00	0.00
D-N-acetylglucosamine	32.00	8.20	0.00	0.00	0.00	4.01	0.00
Methyl-palmitate	57.73	14.36	1.67	0.00	0.00	0.00	0.00
Astaxanthin	0.44	0.11	0.00	0.11	0.00	0.00	0.00
Water	246.01	9.91	204.97	0.00	443.27	0.00	0.09
Carbon dioxide	0.00	0.00	0.00	0.00	0.00	0.00	0.00
Magnesium chloride	0.00	0.00	0.00	0.00	0.00	0.00	0.00
Calcium chloride	0.00	0.00	0.00	0.00	0.00	0.00	0.00
Hydrogen chloride	0.00	0.00	0.00	0.00	0.00	0.00	0.00
Orthophosphoric- acid	0.00	0.00	0.00	0.00	0.00	0.00	0.00
Ethanol	0.00	0.00	0.00	0.00	0.00	0.00	0.00
Sodium hydroxide	0.00	0.00	0.00	0.00	6.88	0.00	0.00
Sodium chloride	0.00	0.00	0.00	0.00	0.00	0.00	0.00
hydroxypropylammoniun	0.00	0.00	0.00	0.00	0.00	0.00	3.25
Sodium acetate	0.00	0.00	0.00	0.00	0.00	0.00	0.00
Sodium metabisulfite	0.00	0.00	0.00	0.00	0.00	0.00	0.00
Sodium hypochlorite	0.00	0.00	0.00	0.00	0.00	0.00	0.00
Acetone	0.00	0.00	0.00	0.00	0.00	0.00	0.00
Total	469.21	48.93	275.80	0.11	460.44	4.01	3.33

**Table 2:** Economic assumptions for the integrated approach of shrimp biorefinery.

Assumptions	Value
Processing capacity (t/y)	4113.09
Main product flowrate (t/y)	2417.66
Raw material cost (USD/t)	6724.27
Plant life (y)	15
Salvage value	10% of depreciable FCI
Construction time of the plant (y)	3
Location	Colombia
Tax rate	39%
Discount rate	8%
Subsidies (USD/y)	0
Type of process	New and unproven
Process control	Digital
Project type	Plant on non-built land
Percentage of contingency	20%
Salary per operator (USD/h)	30
Utilities	Electricity, steam, water
Process fluids	Solid-liquid-gas

**Table 3:** The selling price of products.

Product	Selling Price (USD/t)
Shrimp meat	16,500
Chitin	17,000
Chitosan	35,000
Nitrogenous extract	1000
Astaxanthin	40,000

**Table 4:** Total capital investment for the mass integrated approach for shrimp biorefinery.

Cost of Capital Investment	Total (USD)
Fixed Capital investment (FCI)	2,718,282.60
Working Capital (WCI)	1,359,141.30
Start-up Costs (SUC)	271,828.26

**Table 5:** The annual operating cost for the mass integrated approach for shrimp biorefinery.

Operating Costs	Total (USD/y)
Direct Production Cost (DPC)	28,762,035.14
Fixed Charge (FCH)	330,588.83
Plant Overhead (POH)	336,960.00
General Expenses (GE)	7,357,395.99

**Table 6:** The primary costs for the mass integrated approach for a shrimp biorefinery.

Primary Costs	Total	Unit
Total Capital Investment (TCI)	4.35	MM USD
Operating Cost (OC)	36.79	MM USD/y
Revenues	41.65	MM USD/y

**Table 7:** Economic indicators for the mass integrated approach for shrimp biorefinery.

Economic Indicator	Value	Units
Gross Profit (depreciation not included) (GP)	4.88	MM USD
Gross Profit (depreciation included) (DGP)	4.70	MM USD
Profit After taxes (PAT)	2.87	MM USD
Economic Potential 1 ($/y)	13.99	MM USD/y
Economic Potential 2 ($/y)	13.77	MM USD/y
Economic Potential 3 ($/y)	4.86	MM USD/y
Cumulative Cash Flow	1.12	(1/y)
Payback Period (depreciation included) (DPBP)	6.00	y
ROI	65.88	%
Net present value (NPV)	10.40	MM USD
Annual Cost/Revenue	1.22	

## References

[1] Sagheer F.A., Al-Sughayer M.A., Muslim S., Elsabee M.Z. (2009). Extraction, and characterization of chitin and chitosan from marine sources in Arabian Gulf. Carbohydr Polym..

[2] IMARC Shrimp market: Global industry trends, share, size, growth, opportunity, and forecast 2020-2025. https://www.imarcgroup.com/prefeasibility-report-shrimp-processing-plant.

[3] Zhao D., Huang W.C., Guo N., Zhang S., Xue C., Mao X. (2019). Two-step separation of chitin from shrimp shells using citric acid and deep eutectic solvents with the assistance of microwave. Polymers.

[4] Mao X., Guo N., Sun J., Xue C. (2017). Comprehensive utilization of shrimp waste based on biotechnological methods: A review. J. Clean. Prod..

[5] Pillai C.K.S., Paul W., Sharma C.P. (2009). Chitin and chitosan polymers: Chemistry, solubility, and fiber formation. Prog. Polym. Sci..

[6] Song Z., Li G., Guan F., Liu W. (2018). Application of chitin/chitosan and their derivatives in the papermaking industry. Polymers.

[7] Moreno-Sader K., Meramo-Hurtado S.I., González-Delgado A.D. (2019). Environmental sustainability analysis of chitosan microbeads production for pharmaceutical applications via computer-aided simulation, WAR, and TRACI assessments. Sustain. Chem. Pharm..

[8] Wang W.T., Shu J., Wang X.L., Huang Y., Wang Y.Z. (2010). Dissolution behavior of chitin in ionic liquids. J. Macromol. Sci. Part B Phys..

[9] Majeti M., Kumar R. (2000). Review of chitin and chitosan applications. React. Funct. Polym..

[10] Mujtaba M., Morsi R.E., Kerch G., Elsabee M.Z., Kaya M., Labidi J., Khawar K.M. (2019). Current advancements in chitosan-based film production for food technology; A review. Int. J. Biol. Macromol..

[11] Kaya M., Salaberria A.M., Mujtaba M., Labidi J., Baran T. (2018). An inclusive physicochemical comparison of natural and synthetic chitin films. Int. J. Biol. Macromol..

[12] Akyuz L., Kaya M., Koc B., Mujtaba M., Ilk S., Labidi J., Salaberria A.M., Cakmak Y.S., Yildiz A. (2017). Diatomite as a novel composite ingredient for chitosan film with enhanced physicochemical properties. Int. J. Biol. Macromol..

[13] Rinaudo M. (2006). Chitin and chitosan: Properties and applications. Prog. Polym. Sci..

[14] Sharif R., Mujtaba M., Rahman M., Shalmani A., Ahmad H., Anwar T., Tiachan D., Wang X. (2018). The Multifunctional Role of Chitosan in Horticultural. Molecules.

[15] Gulzar S., Raju N., Chandragiri R., Benjakul S. (2020). Oil and pigments from shrimp processing by-products: Extraction, composition, bioactivities and its application—A review. Trends Food Sci. Technol..

[16] Ambati R.R., Phang S.M., Ravi S., Aswathanarayana R.G. (2014). Astaxanthin: Sources, Extraction, Stability, Biological Activities and Its Commercial Applications—A Review. Mar. Drugs.

[17] Jyonouchi H., Sun S., Iijima K., Gross M.D. (2000). Antitumor activity of astaxanthin and its mode of action. Nutr. Cancer..

[18] Park S.Y., Binkley R.M., Kim W.J., Lee M.H., Lee S.Y. (2018). Metabolic engineering of Escherichia coli for high-level astaxanthin production with high productivity. Metab. Eng..

[19] Abdou E.S., Nagy K.S.A., Elsabee M.Z. (2008). Extraction and characterization of chitin and chitosan from local sources. Bioresour. Technol..

[20] Winsock T., Ghazouani S., Le Bourdieu S. (2019). A methodology for designing thermodynamic energy conversion systems in industrial mass/heat integration problems based on MILP models. Energy..

[21] Barlow J., Sims R.C., Quinn J.C. (2016). Techno-economic and life-cycle assessment of an attached growth algal biorefinery. Bioresour. Technol..

[22] Yu B.Y., Tsai C.C. (2020). Rigorous simulation, and techno-economic analysis of a bio-jet-fuel intermediate production process with various integration strategies. Chem. Eng. Res. Des..

[23] Romero J.C., Vergara L.A., Peralta-Ruiz Y.Y., González-Delgado A.D. (2017). A techno-economic sensitivity approach for development of a palm-based biorefineries in Colombia. Chem. Eng. Trans..

[24] Meramo-Hurtado S.I., Sanchez-Tuiran E., Ponce-Ortega J.M., El-Halwagi M.M., Ojeda-Delgado K.A. (2020). Synthesis and Sustainability Evaluation of a Lignocellulosic Multifeedstock Biorefinery Considering Technical Performance Indicators. ACS Omega.

[25] Nirmal N.P., Benjakul S. (2011). Retardation of quality changes of Pacific white shrimp by green tea extract treatment and modified atmosphere packaging during refrigerated storage. Int. J. Food Microbiol..

[26] Bonfante-Alvarez H., De Avila-Montiel G., Herrera-Barros A., Torrenegra-Alarcón M., González-Delgado A.D. (2018). Valuation of five chitosan production routes with astaxanthin recovery from shrimp exoskeletons. Chem. Eng. Trans..

[27] Dave D., Liu Y., Pohling J., Trenholm S., Murphy W. (2020). Astaxanthin recovery from Atlantic shrimp (Pandalus borealis) processing materials. Bioresour. Technol. Rep..

[28] Srinivasan H., Kanayairam V., Ravichandran R. (2018). Chitin and chitosan preparation from shrimp shells Penaeus monodon and its human ovarian cancer cell line, PA-1. Int. J. Biol. Macromol..

[29] Meramo-Hurtado S., Alarcón-Suesca C., González-Delgado A.D. (2020). Exergetic sensitivity analysis and environmental evaluation of chitosan production from shrimp exoskeleton in Colombia. J. Clean. Prod..

[30] Jane J., Shen L., Wang L., Maningat C.C. (1992). Preparation, and Properties of Small-Particle Corn Starch. Cereal Chem.

[31] Kandra P., Challa M.M. (2012). Efficient use of shrimp waste: Present and future trends. Appl. Microbiol. Biotechnol..

[32] Salman D.D., Ulaiwi W.S., Qais A. (2018). Preparation of chitosan from Iraqi shrimp shell by autoclave, studying some physicochemical properties and antioxidant activity. J. Pharm. Sci. Res..

[33] De Queiroz Antonino R.S.C.M., Lia Fook B.R.P., De Oliveira Lima V.A., De Farias Rached R.Í., Lima E.P.N., Da Silva Lima R.J., Peniche Covas C.A., Lia Fook M.V. (2017). Preparation and Characterization of Chitosan Obtained from Shells of Shrimp (Litopenaeus vannamei Boone). Mar. Drugs..

[34] Gonzalez Bell J. (2019). Producción local de camarón. La República. https://www.agronegocios.co/agricultura/produccion-local-de-camaron-completo-cuatro-anos-al-alza-aumento-de-21-comparado-con-2017-2827251.

[35] Herrera-Rodriguez T., Parejo-Palacio V., González-Delgado A.D. (2018). Technoeconomic sensitivity analysis of industrial agar production from red algae. Chem. Eng. Trans..

[36] El-Halwagi M.M. (2012). Overview of Process Economics. Sustain. Des. Through Process Integr..

[37] Perez Zúñiga D.L., Luna Barrios E.J., Peralta-Ruiz Y.Y., González-Delgado A.D. (2016). Techno-economic sensitivity of bio-hydrogen production from empty palm fruit bunches under colombian conditions. Chem. Eng. Trans..

[38] Alibaba.com: Manufacturers, Suppliers, Exporters & Importers from the world’s largest online B2B marketplace. https://spanish.alibaba.com/.

[39] Peters M.S., Timmerhaus K.D., West R. (2003). Plant Design and Economics for Chemical Engineers.

[40] El-Halwagi M.M. (2012). Sustainable Design through Process Integration: Fundamentals and Applications to Industrial Pollution Prevention, Resource Conservation, and Profitability Enhancement.

[41] Ninõ-Villalobos A., Puello-Yarce J., González-Delgado A.D., Ojeda K.A., Sánchez-Tuirán E. (2020). Biodiesel and Hydrogen Production in a Combined Palm and Jatropha Biomass Biorefinery: Simulation, Techno-Economic, and Environmental Evaluation. ACS Omega..

[42] Gómez-Ríos D., Barrera-Zapata R., Ríos-Estepa R. (2017). Comparison of process technologies for chitosan production from shrimp shell waste: A techno-economic approach using Aspen Plus®. Food Bioprod. Process..

[43] Zuorro A., Lavecchia R. (2011). Polyphenols and energy recovery from spent coffee grounds. Chem. Eng. Trans..

[44] Zuorro A., Lavecchia R., Medici F., Piga L. (2014). Use of cell wall degrading enzymes for the production of high-quality functional products from tomato processing waste. Chem. Eng. Trans..

[45] Zuorro A. (2014). Response surface methodology analysis of polyphenol recovery from artichoke waste. Am. J. Appl. Sci..

[46] Zuorro A., Lavecchia R., Maffei G. (2015). Enhanced lipid extraction from unbroken microalgal cells using enzymes. Chem. Eng. Trans..

[47] Panusa A., Petrucci R., Lavecchia R., Zuorro A. (2017). UHPLC-PDA-ESI-TOF/MS metabolic profiling and antioxidant capacity of arabica and robusta coffee silverskin: Antioxidants vs phytotoxins. Food Res. Int..

[48] Cogollo-Herrera K., Bonfante-Álvarez H., De Ávila-Montiel G. (2018). Herrera- Barros, A.; González-Delgado, A.D.Techno-economic sensitivity analysis of large scale chitosan production process from shrimp shell wastes. Chem. Eng. Trans..

